# Magnetic resonance enterography changes after antibody to tumor necrosis factor (anti-TNF) alpha therapy in Crohn’s disease: correlation with SES-CD and clinical-biological markers

**DOI:** 10.1186/s12880-016-0139-7

**Published:** 2016-05-05

**Authors:** Luca Pio Stoppino, Nicola Della Valle, Stefania Rizzi, Elsa Cleopazzo, Annarita Centola, Donatello Iamele, Christos Bristogiannis, Giuseppe Stoppino, Roberta Vinci, Luca Macarini

**Affiliations:** Division of Diagnostic Imaging, Department of Surgical Sciences, University of Foggia, Viale Luigi Pinto n.1, Foggia, 71122 Italy; Division of Gastroenterology, Department of Surgical Sciences, University of Foggia, Viale Luigi Pinto n.1, Foggia, 71122 Italy; Division of Gastroenterology, Department of Surgical Sciences, Azienda Sanitaria Locale Provincia di Foggia, Piazza della Libertà n.1, Foggia, 71122 Italy

**Keywords:** Crohn’s disease, Magnetic resonance enterography, Anti-TNF drugs, Simple Endoscopic Score for Crohn’s Disease

## Abstract

**Background:**

In recent years, the use of MRI in patients with Crohn’s disease (CD) has increased. However, few data are available on how MRI parameters of active disease change during treatment with anti-TNF and whether these changes correspond to symptoms, serum biomarkers, or endoscopic appearance. The aim of this study was to determine the changes over time in MRI parameters during treatment with anti-TNF in patients with CD, and to verify the correlation between MRI score, endoscopic appearance and clinical-biological markers.

**Methods:**

We performed a prospective single centre study of 27 patients with active CD (18 males and 9 females; median age of 27,4 ys; age range, 19–49). All patients underwent ileocolonoscopy and MRI at baseline and 26 weeks after anti-TNF therapy. Endoscopic severity was graded according to the Simple Endoscopic Score for Crohn’s Disease (SES-CD) and Magnetic Resonance Index of Activity (MaRIA) was calculated. Patients underwent clinical evaluation (CDAI) and the C-reactive protein (CRP) level was measured. The associations between variables were assessed with Pearson’s bivariate correlation analysis.

**Results:**

A total of 135 intestinal segments were studied. The median patient age was 27,4 years, 67 % were male and the mean disease duration was 6,1 years. For induction of remission, 18 patients were treated with infliximab and 9 with adalimumab. The mean SES-CD and MaRIA scores significantly changed at week 26 (SES-CD: 14,7 ± 8,9 at baseline vs. 4,4 ± 4,6 at 26 weeks - *p* < 0.001; MaRIA: 41,1 ± 14,8 at baseline vs. 32,8 ± 11,7 at 26 weeks - *p* < 0.001). Also the CDAI and serum levels of CRP decreased significantly following treatment (*p* < 0.001). The overall MaRIA correlated with endoscopic score and with clinical activity (CDAI) both at baseline and at week 26 (*p* < 0.05). The correlation between overall MaRIA and CRP was significant only at week 26 (*p* < 0.001).

**Conclusions:**

The MaRIA has a good correlation with SES-CD, a high accuracy for prediction of endoscopic mucosal healing and is a reliable indicator to monitor the use of TNF antagonists in patients with CD.

## Background

Crohn’s disease (CD) is a disabling transmural and segmental chronic inflammatory bowel disease (IBD) with a relapsing and remitting course. Its inflammatory lesions can affect the entire gastrointestinal (GI) tract leading to various intestinal (internal and external fistulas, intestinal strictures, abdominal and perianal abscesses) and extra-intestinal manifestations [1].

Although its aetiology is still unknown considerable progress has been made in the understanding of the molecular mediators and mechanisms of tissue injury. Current treatment protocols, based on the use of drugs with a gradually increasing strength of action, are aimed at modulating the complex inflammatory events leading to intestinal injury [2].

The proinflammatory cytokine Tumor Necrosis Factor (TNF) alpha is a key mediator of inflammation associated with CD [3] and the recent development of antibody to TNF alpha (anti-TNF) drugs has led to significant improvements in the medical treatment of these patients [4, 5].

These antagonists of TNF-alpha, i.e. infliximab (IFX) and adalimumab (ADA), are effective in inducing as well as maintaining clinical remission in patients with moderately-to-severely active CD disease who are refractory to traditional treatments (corticosteroids and immunosuppressive drugs). Complete disappearance of mucosal ulcerations is associated with favourable outcome, and after initiation with anti-TNF, mucosal healing (MH) leads to a decrease both in relapse rates and in disease related hospitalization, reducing the need for surgery [6].

Unfortunately, the proven clinical efficacy of anti-TNF drugs is contrasted with the elevated frequency of premature relapses on discontinuing treatment once maintained remission of the disease is achieved [7]. This phenomenon is attributed to the persistence of inflammatory activity despite an apparent positive clinical response [8]. In fact, CD is a typically transmural disease and its activity can be difficult to accurately evaluate. Therefore, for assessing CD activity, for tailoring therapy, and for measuring treatment response, objective determination of inflammatory activity should be essential. The gold standard for assessment of luminal inflammation in CD is endoscopy with biopsies, which can evaluate MH but it is invasive, exposes patients to inherent procedural risks, and is unable to assess the mid-small intestine [9]. Symptom-based disease activity indexes are subjective by design and often unreliable [10].

Cross-sectional imaging can serve as an alternative or an adjunct to ileocolonoscopy to evaluate therapeutic response and transmural healing. Computed tomographic and magnetic resonance enterography have been reported to be useful modalities for the evaluation of luminal inflammation and extra intestinal complications in CD. MRI can be performed without radiation exposure, making it the preferred imaging technique for the evaluation of CD [11]. To our knowledge the effects of the biological agents on transmural inflammation and their resulting imaging are largely unknown.

The primary aim of this study was to, therefore, determine the changes over time in MRI parameters during treatment with anti-TNF in patients with CD. Secondary aim is to examine whether radiologic response to anti-TNF treatment correlates with endoscopic appearance and clinical-biological markers.

## Methods

### Patients

Between April 2012 and April 2015, 27 outpatients diagnosed with CD according to the Lennard-Jones criteria (Table 1) [12], were prospectively studied at single centre. The patient cohort comprised 18 males and 9 females, with a median age of 27,4 years (age range, 19–49). The median disease duration was 6,1 years (mean SD, 2,2). Inclusion criteria were age ≥ 18, moderate-to-severe intestinal disease (Crohn’s Disease Activity Index – CDAI - score > 220 points) and elevated C-reactive protein (CRP) level (**>**5 mg/l). Exclusion criteria were active or latent tuberculosis, contraindications for MR, treatment with more than 15 mg of systemic corticosteroids (prednisone equivalent) within the 2 weeks prior to baseline MRI, documented abdominal abscess or internal fistula as well as medical contraindications for anti TNF therapy.

**Table 1:** Lennard-Jones anatomic criteria for the diagnosis of CD recognizable by clinical, radiological and pathologic examination

	Clinical/endoscopy	X-ray	Biopsy	Resected specimen
Mouth to anus				
Upper gut	+	+	+	+
Anus	+		+	+
Discontinuous	+	+	+	+
Transmural inflammation				+
Fissure		+		+
Abscess	+	+		+
Fistula	+	+		+
Fibrosis/Stenosis	+	+		+
Lymphoid				
Ulcers			+	+
Aggregates			+	+
Mucin Retention			+	
Granuloma			++	++

^a^A diagnosis of Crohn’s disease requires 3 positive findings, or one positive finding with granulomas on histology

The anti-TNF treatment consisted of administering an induction regimen, either with IFX or ADA in the case of patients who had previously been treated with IFX and who had presented complications in its administration. The induction regimen for IFX consisted of administering three intravenous doses of 5 mg/Kg in weeks 0, 2 and 6, and maintenance every 8 weeks thereafter. The induction regimen for ADA consisted of an 160 mg subcutaneous injection as an initial dose, followed by 80 mg after 2 weeks and 40 mg every other week thereafter [5]. After obtaining written informed consent, endoscopy (reference standard), clinical-biological assessment and MRI were performed in all patients prior to the first anti-TNF drugs infusion and at week 26. Ileocolonoscopy and MRI were performed within a maximum interval of 7 days and the time gap between these tests and the start-end of anti-TNF therapy was to a maximum of 3 days.

### Endoscopic examination

Ileocolonoscopy was considered the reference standard for the evaluation of IBD extension and severity. In all cases endoscopy was performed under anaesthesia by experienced endoscopist in IBD (NDV) using standard equipment (CV-180; Olympus, Japan) and following the standard protocol used in clinical practice (colonic cleansing with 4 L polyethylene glycol plus low-fiber diet 3 days before). The length of terminal ileum evaluated on colonoscopy ranged from 5 to 15 cm. Quantification of endoscopic lesions was calculated globally and per segment using the Simple Endoscopic Score for Crohn’s Disease (SES-CD). For accuracy of endoscopic data collection, endoscopist completed the SES-CD on a predefined scoring sheet immediately after finishing the procedure.

For the grading of endoscopic findings with SES-CD, the bowel was divided into 5 segments: terminal ileum; right, transverse, and left colon; and rectum. Four endoscopic variables in the 5 segments were scored from 0 to 3 [13]. The variable “presence and size of ulcers” was scored 0 when no ulcers were present, 1 for small ulcers (diameter, 0.1**–**0.5 cm), 2 for medium-sized ulcers (diameter, 0.5**–**2 cm), and 3 for large ulcers (**>**2 cm). The variable “extent of ulcerated surface” was scored 0 when no ulcers were present, 1 when extent was **<**10 %, 2 when extent was 10 % to 30 %, and 3 when extent was **>**30 %. The variable “extent of affected surface” was scored 0 if none, 1 when **<**50 %, 2 when 50 % to 75 %, and 3 when **>**75 %. The variable “presence and type of narrowing” was scored 0 when no narrowing was present, 1 for a single passable narrowing, 2 for multiple passable narrowed areas, and 3 for a non-passable narrowing. The resulting score was (Table 2): SES-CD = sum of all variables – 1,4 × number of affected segments.

**Table 2:** Scoring sheet for SES-CD

	Ileum	Right colon	Transverse colon	Left colon	Rectum	SUM
Presence and size of ulcers						+
Extent of ulcerated surface						+
Extent of affected surface						+
Presence and type of narrowing						=
Sum of all variables						TOT
Affected segments	☐	☐	☐	☐	☐	

TOT – 1.4 × (number of affected segments) = SES-CD

The SES score can range from 0 to 60, with a higher score indicating more severe disease. Investigator reporting the endoscopic lesions was blinded to the results of the MRI examination.

### Magnetic resonance enterography

All MRI examinations were performed in the supine position with a 1.5 T magnet (Achieva, Philips Medical System, Eindhoven, The Netherlands) equipped with a phased-array-16-elements coil. 1 h prior to MRI, all patients received orally 1000–1500 ml of iso-osmotic PEG solution, which was freshly prepared by dissolving in water a granular powder containing PEG (58.32 g), sodium sulphate (5.69 g) sodium bicarbonate (1.69 g), sodium chloride (1.46 g) and potassium chloride (0.74 g) (Selg 1000, Promefarm, Milan, Italy). In order to ensure an adequate intestinal distension, a coronal T2 scan was performed after 30 min after oral contrast administration. If the minimum diameter of the small bowel loops was 15 mm o larger, the bowel distension was judged satisfactory and MRI was continued after intravenous administration of 20 mg N-butylscopolamine (Buscopan, Boringher Ingelheim, Ingelheim am Rhein, Germany) in order to suppress small bowel peristalsis and avoid motion artifacts.

Then, the acquisition protocol outlined in Table 3 was performed. 3D T1-weighted high-resolution isotropic volume excitation (THRIVE) before and 30–40s, 70–90s and 120–140 s after intravenous administration of 0.2 ml/kg body weight of gadolinium chelate (Gd-DTPA, Magnevist, Schering AG, Germany) and finally a T1-weighted water selective (WATS) fat-saturated sequence in the axial plane late after injection were acquired.

**Table 3:** MRE protocol

	T2-TSE	T2 SPAIR	DUAL FFE	BFFE	Gd-DTPA THRIVE	T1 WATS
Plane	Axial	Axial	Axial	Coronal	Coronal (3D)	Axial
Slices thickness (mm)	5	5	5	4	1.5	5
FOV (mm)	450x450	400x400	450x450	400x400	420x420	450x450
TR (ms)	1200	1200	140	3,7	2.3	346
TE (ms)	80	80	4,6/2,3	1,9	4.7	6.6
Flip angle	90	90	80	40	10	70

Image analysis was performed by one experienced radiologist (LPS) and one junior radiologist (SR) using a dedicated postprocessing workstation (ViewForum, Philips Medical System, Eindhoven, The Netherlands). To allow comparison with endoscopic score, the same division into segments was considered. The small and large bowel were examined to detect the segment with the most severe lesions on the basis of the following criteria: bowel wall thickness (mm), presence of mucosal ulcers (defined as deep depressions in the mucosal surface), presence of mural oedema (hyperintensity on T2-weighted sequences of the bowel wall relative to the signal of the psoas muscle), presence of enlarged regional mesenteric lymph nodes, presence of fistula or abscess, and relative contrast enhancement (RCE) of the intestinal wall. Quantitative measurements of wall signal intensity (WSI) were obtained from the areas with the greatest thickening [region of interest (ROI)] before and after intravenous injection of gadolinium (70 s). RCE was calculated according to the following formula: RCE = [(WSI postgadolinium − WSI pregadolinium)/(WSI pregadolinium)] × 100 × (SD noise pregadolinium/SD noise postgadolinium). As defined by Rimola et al. [14, 15] for measurement of therapeutic response by means of MRI and to allow comparison with the reference standard (SES-CD), the MaRIA in each segment was calculated according to the following formula: 1.5 × wall thickening (mm) + 0.02 × RCE (relative contrast enhancement) + 5 × oedema + 10 × ulcers.

The global MaRIA score was calculated as the sum of the MaRIA in ileum, right colon, transverse colon, left colon-sigmoid and rectum.

### Response assessment

In order to quantify disease activity the SES-CD and the MaRIA were calculated at baseline and 26 weeks after treatment initiation. MH was defined as the absence of mucosal ulcerations at week 26 in patients who had mucosal lesions endoscopically confirmed at baseline [16]. The endoscopic response was defined as a decrease from baseline in SES-CD score of at least 5 points and at least 50 % [17] with a complete endoscopic remission (MH) when SES-CD score ≤ 2 [18]. In MRI examinations the MH was defined as the complete disappearance of intestinal lesions at week 26, while the radiologic response was defined as an overall MaRIA score reduction of at least 9.7 points.

In addition, the response to treatment was assessed both clinically as well as biologically by calculating the CDAI and the nephelometric determination of the serum concentration of CRP. The CDAI is a numerical calculation derived from the sum of products from a list of 8 items (Table 4), and multiplied by weighting factors for each item to define the severity of “disease activity” in patients with CD [19]. Three categories were identified to define the clinical-biological response: (a) Lack of response when the CDAI and CRP levels increased or did not change; (b) Partial response when the CDAI decreased by more than 70 points and CRP levels decreased but did not restore to normal and (c) Remission when the CDAI was lower than 150 points and CRP levels were normal.

**Table 4:** CDAI items and weighting factors

Item (daily sum per week)	Weighting factor
Number of liquid or very soft stools	2
Abdominal pain score in one week (rating, 0–3)	5
General well-being (rating, 1–4)	7
Sum of physical findings per week:	20
Arthritis/arthralgia	
Mucocutaneous lesions (e.g. erythema nodosum, aphthous ulcers)	
Iritis/uveitis	
Anal disease (fissure, fistula, etc.)	
External fistula (enterocutaneous, vesicle, vaginal, etc.)	
Fever over 37.8 °C	
Antidiarrheal use	30
Abdominal mass (no = 0, equivocal = 2, yes = 5)	10
47 minus hematocrit (males) or 42 minus hematocrit 6 (females)	6
1-x (1-body weight divided by a standard weight)	1

### Statistical analysis

Quantitative variables are given as means and standard deviation (SD) and proportions are expressed as percentages and 95 % confident intervals (CIs). Differences in quantitative measures were tested by Student’s test. The associations between continuous variables were evaluated with Pearson’s bivariate correlation analysis. To determine the best cut-off value both of the overall MaRIA and of the Δ MaRIA scores for predicting endoscopic remission (SES-CD score ≤ 2) receiver operating characteristic (ROC) curves were calculated. Inter-observer agreement between paired evaluations of MR by two radiologists (LPS and SR) was performed with Pearson correlation coefficient.

The Statistical Package for the Social Sciences (version 21, SPSS Inc, Chicago, Ilinnois, USA) was used to describe and analyse the data, considering values of *p* < 0.05 as significant.

## Results

27 consecutive patients were included in the study for a total of 135 segments explored by ileocolonoscopy and then evaluated by MRI. Baseline characteristics of the patients are given in Table 5. Before the administration of the anti-TNF treatment, all patients had a CDAI > 220 points, a CRP level greater than 5 mg/l and a SES-CD > 3. For induction of remission, 18 patients (67 %) were treated with IFX and 9 (33 %) with ADA.

**Table 5:** Baseline characteristics of the patients

Patients *n* = 27	
M/F	18/9
Age at diagnosis [median]	27,4
Disease duration [years, mean (SD)]	6,1 (2,2)
Disease location	
-Rectum	0
-Sigmoid/Left colon	9
-Transverse colon	3
-Right colon	3
-Ileum	27
Anti-TNF drugs	
-IFX	18
-ADA	9

**Fig. 1:**
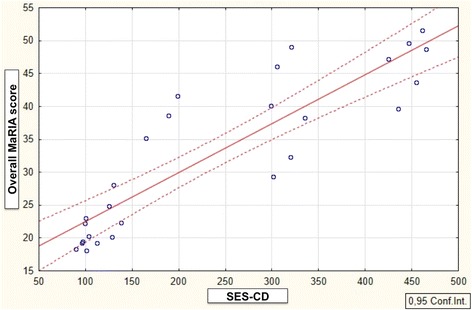
Correlation between overall MaRIA score and SES-CD at week 26

The correlation between overall SES-CD and overall MaRIA was good at baseline (*p* = 0.03) and very high at week 26 (*p* < 0.001) (Fig. 1).

A significant correlation between overall MaRIA and CDAI, including both baseline and week 26, was observed (*p* = 0.03; *p* < 0.001, respectively). The correlation between overall MaRIA and CRP was significant only at week 26 (baseline: *p* = 0.4; week 26: *p* < 0.001). A significant correlation of the Δ MaRIA score was observed with Δ SES-CD (*p* < 0.001), Δ CDAI (*p* < 0.001) and Δ CRP (*p* < 0.001).

The administration of the anti-TNF drug induced endoscopic response in 16 patients (59 %) and among these a complete disease remission/MH (SES-CD ≤ 2) occurred in 13 patients (48 %). 10 patients (37 %) showed a stable or slightly lower SES-CD compared to baseline. Only 1 patient (4 %) had a SES-CD slightly increased at the end of the study.

**Fig. 2:**
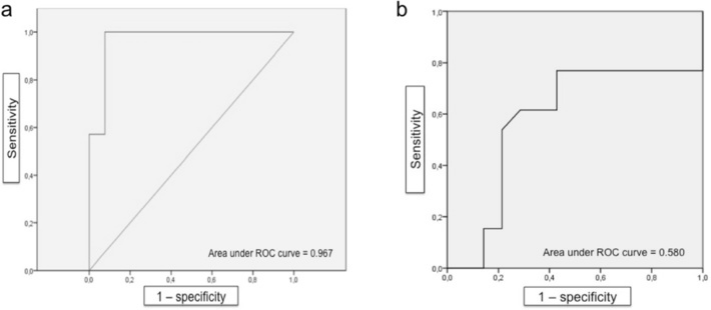
Receiver operating characteristic (ROC) curves of the overall MaRIA (**a**) and of Δ MaRIA (**b**) scores to predict endoscopic remission/MH

**Fig. 3:**
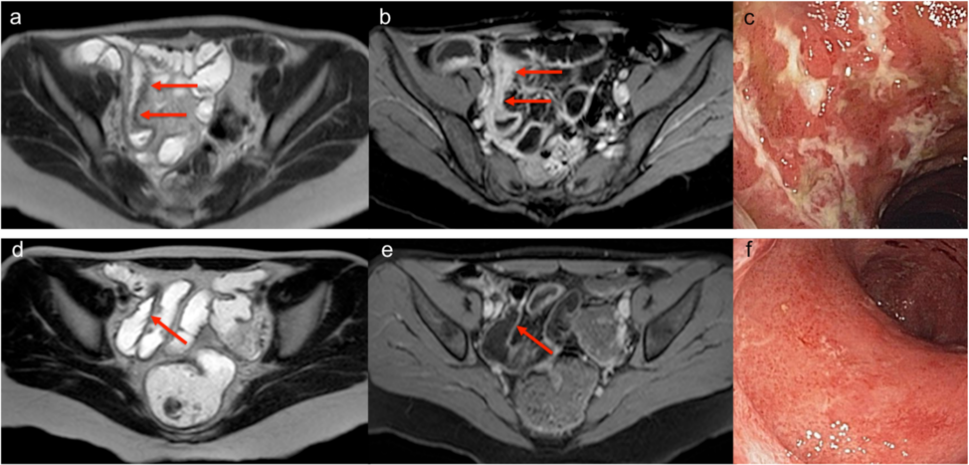
Patient with CD of the terminal ileum in treatment with IFX. At baseline, MRI (**a** and **b**) detected moderate inflammatory lesions of the terminal ileum, with wall thickening accompanied by oedema, irregularity of the mucosal surface and hyperenhancement after intravenous contrast administration (Overall MaRIA score = 49). Baseline endoscopy of the same segment confirmed the presence of serpiginous, longitudinal ulcerations (**c**; SES-CD = 20). At week 26, the terminal ileum achieved healing at both MRI (**d** and **e**; Overall MaRIA score = 19,2; Δ MaRIA score = 29,8) and endoscopy (**f**; SES-CD = 0)

Using a cut-off point of 30.8 the overall MaRIA was found to have a high accuracy for prediction of endoscopic MH (SES-CD score ≤ 2) with an area under the ROC curve of 0.967, sensitivity of 93 % and specificity of 77 % (Fig. 2). At week 26 the overall MaRIA score was **<** 30.8 in 13 patients (48 %; Fig. 3).

**Fig. 4:**
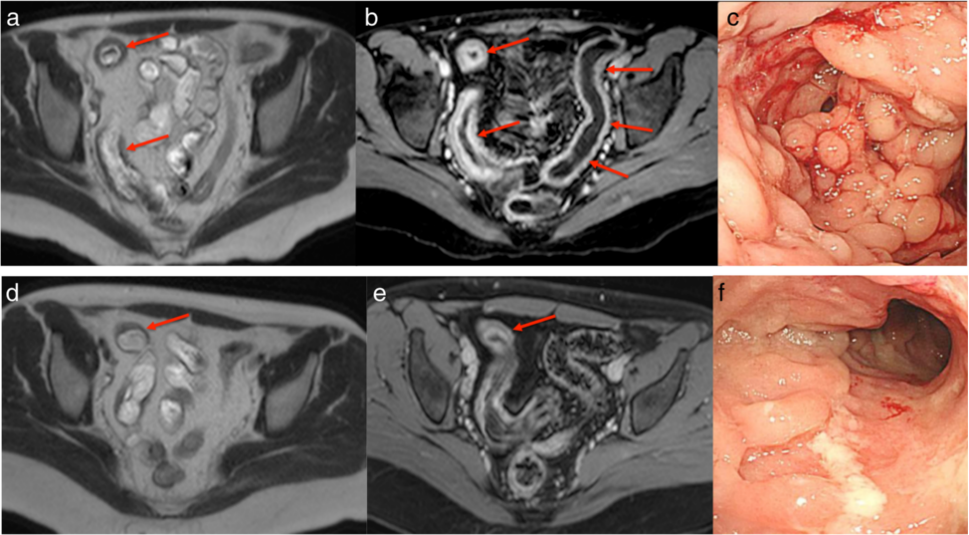
Patient with CD of the distal ileum, left and transverse colon in treatment with IFX. At baseline, MRI (**a** and **b**) detected severe inflammatory lesions of the terminal ileum, with marked wall thickening accompanied by oedema, extensive irregularity of the mucosal surface and stratified hyperenhancement after intravenous contrast administration. A moderate wall thickening of the left colon is also present with hyperenhancement after intravenous contrast administration (Overall MaRIA score = 62,7). Baseline endoscopy at the distal ileum revealed the presence of stricturing, cobblestone appearance of the mucosal surface (**c**; SES-CD = 33). At week 26, the distal ileum continues to present at MRI a moderate wall thickening with mild hyperenhancement after intravenous contrast administration (**d** and **e**; Overall MaRIA score = 52,6; Δ MaRIA score = 10,1) Endoscopy of the same segment shows irregular longitudinal ulcers (**f**; SES-CD = 10)

A Δ MaRIA score ≥ 9.7 had a good diagnostic accuracy for predicting endoscopic remission/MH with sensitivity of 77 % and specificity of 57 % (area under the curve 0.580; 95 % CI: 0.634–0.944). At week 26 the overall MaRIA score decreased of at least 9.7 points in 10 patients (37 %) while increased in 4 patients (15 %). In the remaining 13 patients (48 %) the overall MaRIA score decreased by less than 9.7 points compared to baseline (Fig. 4).

Clinical-biological assessment of therapeutic response demonstrated that a CDAI < 150 at week 26 was achieved in 12 patients (44 %) and CRP levels restored to normal in 21 patients (78 %). At week 26, CRP levels reduced in 6 patients (22 %) and 3 patients (12 %) had a CDAI decrease of more than 70 points, defined as a partial response. Lack of response with CDAI increased or not changed was observed in remaining 12 patients (44 %). No patients showed an increase of CRP levels.

The results of the endoscopic, MRI and clinical-biological changes induced by the treatment are shown in Table 6.

**Table 6:** Endoscopic, MRE, clinical and biological changes induced by anti-TNF treatment

	Pre-treatment	Post-treatment
SES-CD [mean (SD)]	14,7 (8,9)	4,4 (4,6)
Overall MaRIA [mean (SD)]	41,1 (14,8)	32,8 (11,7)
CDAI [mean (SD)]	423,7 (71,1)	238,5 (140,1)
CRP [(mg/l) mean (SD)]	25,1 (23,6)	4,6 (5,6)

In terms of endoscopy and MRI, there was a statistically significant reduction both in SES-CD and in MaRIA score (*p* < 0.001). Also the CDAI and serum levels of CRP decreased significantly following treatment (*p* < 0.001).

We observed high interobserver agreement for the overall MaRIA score both at baseline and at week 26 (κ = 0.93, s.e. = 0.88; κ = 0.95, s.e. = 0.98, respectively). Accuracy rates for the overall MaRIA score were 92 % at baseline and 96 % at the end of the study.

## Discussion

Since the beginning of anti-TNF therapy, MH has become an important predictor of long-term disease outcome in IBD. MH during scheduled anti-TNF therapy reduces need for surgery and hospital treatment significantly [20]. Accordingly, the assessment of therapeutic efficacy requires close monitoring of the mucosa and the bowel wall. To date, ileocolonoscopy remains the gold standard for assessing disease remission in CD, and in the present study we prefer to use the SES-CD since it is a reproducible index for this evaluation [13, 21], easier and faster to calculate than Crohn’s Disease Endoscopic Index of Severity (CDEIS). In fact CDEIS, although it has proven to be a reliable and reproducible marker of MH in a number of therapeutic trials [22–24], is rather time consuming and elaboration of the score requires analogue scale transformation. These characteristics make CDEIS unsuitable for everyday clinical practice and also its use can be complex in clinical trials. Nevertheless, endoscopy remains an invasive procedure with potential complications [25], can be felt as a problem by CD patients and does not reflect the overall burden of the disease [26].

As the small bowel is difficult to reach with conventional endoscopy, several non-invasive tools have been developed in the last two decades in the investigation of CD. MRI is probably to date the best alternative, owing to its nonionizing characteristics and high performances especially in detecting signs of intestinal inflammation [27]. In particular, MRI has great potentials in characterization of the CD, being able to assess parameters such as parietal thickening, hypervascularity (comb signs), mesenteric fibro-fatty proliferation and others [11]. Since CD is a trans-mural disease, the use of MRI represent an important step in the diagnostic, therapeutic and prognostic management of the disease, because the mucosal lesions assessable by endoscopy represents only the tip of the inflammatory process. These findings call for revision of the current understanding of “intestinal healing” in the treatment of CD. MRI may be in fact employed to determine microscopic structural wall changes, including edema and fibrosis, hypervascularity, capillary permeability, and likely, in the next years, specific molecular abnormalities, extending beyond the concept of “mucosal healing” [27].

Recently Rimola and co-workers developed the MaRIA score, which is able to assess inflammation in ileal and colonic CD [14, 15]. The MaRIA score is a validated index for describing the severity of inflammation, but it is not a gold standard for describing CD, which is much more complex. Despite this limitation, MRI in CD can be considered as the most validated tool for evaluating inflammation.

In our study, we evaluated MRI findings, before and after medical therapy with anti-TNF, and the correlation between endoscopic assessments, clinical-biological responses and MRI modifications. The first study assessing the responsiveness of MRI in patients with CD was published in 1999 [28]. In this small study, 8 patients with active CD were examined before and after treatment with corticosteroids using low-field magnetic resonance (1.0 T). The MRI parameters that showed a significant reduction during treatment were contrast enhancement (*p* < 0.001) and wall thickness (*p* = 0.03), which is in agreement with the results of the current study.

More recently, Ordas et al. [29] performed a prospective multicentre study of 48 patients with active CD, examining patients who underwent ileocolonoscopy and MRI at baseline and 12 weeks after treatment with steroid or tumor necrosis factor inhibitor (specifically ADA). The primary end points for the study were determining the accuracy of MRI in identifying ulcer healing (defined as the disappearance of ulcers on endoscopic exam) and the endoscopic remission (quantified using CDEIS <3.5). MRI had a high diagnostic accuracy for both predicting endoscopic ulcer healing, with sensitivity of 75 % and specificity of 80 %, and endoscopic remission, with sensitivity of 83 % and specificity of 84 %. Our data showed similar MRI diagnostic accuracy for predicting endoscopic remission/MH with a higher sensitivity (93 %) and a slightly lower specificity (77 %). These comparable results were obtained in spite of our study was a single centre using a 1.5 T MR unit while the Ordas was a multicentre study performed with 3.0 T scanner, which should have a better signal and higher spatial resolution. Moreover, the results were not influenced despite the use of different endoscopic indexes, different therapeutic strategies and the lack of colon distension by instillation of water through a rectal catheter.

Similar to our study, Van Assche and co-workers [30] recruited only anti**-**TNF-naïve patients in a multicentric and prospective trial evaluating the effects of IFX therapy on MRI ileal CD lesions. This pilot study included 15 patients that were studied at baseline, 2, and 26 weeks after starting IFX induction and maintenance therapy. The authors concluded that normalization of MRI findings is rare after IFX therapy. This is apparently discrepant with the observations of the current study, in which normalization of MRI findings paralleled endoscopic and clinical responses. The reasons for this discrepancy might include the fact that the study by Van Assche et al. used a different activity index and it only assessed ileal disease in a limited sample size.

In the current study a prospective series of patients with ileo-colonic CD was assessed in which a 59 % rate of endoscopic partial or complete response was achieved using an anti-TNF induction therapy. This percentage is similar to those described in the literature [31]. In our series, a reduction of the overall MaRIA score was found in 48 % of patients and a significant improvement in Δ MaRIA score was found in 37 % of patients following the treatment. A complete disappearance of MRI alterations was found only in 5 patients. As expected, the MRI improvement was significantly related to the endoscopic and the clinical-biological response in such a way that it only occurred in patients who responded to the treatment. These data support the reliability of MRI as a tool in assessing response to treatment in-patient with CD.

This study has its limitations. In a purely observational design, our endoscopic end-points MRI, laboratory and clinical findings present the outcome of a heterogeneous CD patient group during treatment with anti-TNF in real-life clinical practice. Confounding relevant factors are our use of both ADA and IFX and their varying dosages and the small proportion of CD patients.

## Conclusions

In conclusion, our data demonstrate that MaRIA have a high correlation both with SES-CD and with clinical-biological activity of the disease. According to Tielbeek et al. [32], MRI is a valid and reliable technique to monitor the use of TNF antagonists in clinical practice and it provides an accurate measure for prediction of endoscopic MH in patients with CD.

### Ethics approval and consent to participate

The protocols were reviewed and approved by the Ethics Committee of our University Hospital and was conducted in accordance with the Declaration of Helsinki. All patients gave written informed consent to participate in this study.

### Availability of supporting data

Due to statutory provisions regarding data- and privacy protection, the dataset supporting the conclusions of this article is available upon individual request directed to the corresponding author.

## Abbreviations

ADA: adalimumab
anti-TNF: anti-tumour necrosis factor-α antibodies
CD: Crohn’s disease
CDAI: Crohn’s Disease Activity Index
CDEIS: Crohn’s Disease Endoscopic Index of Severity
CI: confidence interval
CRP: C-reactive protein
CTE: computed tomography enterography
IBD: inflammatory bowel disease
IFX: infliximab
IL: interleukin
MH: mucosal healing
MRI: magnetic resonance imaging
PEG: polyethylene glycol
RCE: relative contrast enhancement
ROC: curve receiver operator characteristic curve
SD: standard deviation
SES-CD: Simple Endoscopic Score for Crohn’s Disease
TNFα: tumour necrosis factor-α
WSI: wall signal intensity

## References

[1] Baumgart DC, Sandborn WJ (2007). Inflammatory bowel disease: clinical aspects and established and evolving therapies. Lancet.

[2] Travis SP, Stange EF, Lemann M (2006). European Crohn’s and Colitis Organisation. European evidence-based consensus on the diagnosis and management of Crohn’s disease: current management. Gut.

[3] Breese E, McDonald TT (1995). TNF alpha secreting cells in normal and diseased human intestine. Adv Exp Med Biol.

[4] Rutgeerts P, Van Assche G, Vermeire S (2004). Optimizing anti-TNF treatment in inflammatory bowel disease. Gastroenterology.

[5] Clark M, Colombel JF, Feagan BC (2007). American Gastroenterological association consensus development conference on the use of biologics in the treatment of inflammatory bowel disease, june 21–23, 2006. Gastroenterology.

[6] Rutgeerts P, Feagan BG, Lichtenstein GR (2004). Comparison of scheduled and episodic treatment strategies of infliximab in Crohn’s disease. Gastroenterology.

[7] Dome’nech E, Hinojosa J, Nos P (2005). Clinical evolution of luminal and perianal Crohn’s disease after inducing remission with infliximab: how long should patients be treated?. AlimentPharmacol Ther.

[8] Lichtenstein GR, Abreu MT, Cohen R, Tremaine W (2006). American Gastroenterological Association Institute technical review on corticosteroids, inmunomoduladors, and infliximab in inflammatory bowel disease. Gastroenterology.

[9] Terheggen G, Lanyi B, Schanz S (2008). Safety, feasibility, and tolerability of ileocolonoscopy in inflammatory bowel disease. Endoscopy.

[10] Freeman HJ (2010). Limitations in assessment of mucosal healing in inflammatory bowel disease. World J Gastroenterol.

[11] Macarini L, Stoppino LP, Centola A (2013). Assessment of activity of Crohn’s disease of the ileum and large bowel: proposal for a new multiparameter MR enterography score. Radiol Med.

[12] Lennard Jones JE (1989). Classification of inflammatory bowel disease. Scand J Gastroenterol.

[13] Daperno M, D’Haens G, Van Assche G (2004). Development and validation of a new, simplified endoscopic activity score for Crohn’s disease: the SES-CD. Gastrointest Endosc.

[14] Rimola J, Rodriguez S, Garcia-Bosch O (2009). Magnetic resonance for assessment of disease activity and severity in ileocolonic Crohn’s disease. Gut.

[15] Rimola J, Ordas I, Rodriguez S (2011). Magnetic resonance imaging for evaluation of Crohn’s disease: validation of parameters of severity and quantitative index of activity. Inflamm Bowel Dis.

[16] Colombel JF, Sandborn WJ, Reinisch W (2010). Infliximab, azathioprine, or combination therapy for Crohn’s disease. N Engl J Med.

[17] Ferrante M, Noman M, Vermeire S (2010). Evolution of endoscopic activity scores under placebo therapy in Crohn’s disease. Gastroenterology.

[18] Moskovitz DN, Daperno M, Van Assche G (2007). Defining and validating cut-offs for the simple endoscopic score for Crohn’s disease. Gastroenterology.

[19] Winship DH, Summers RW, Singleton JW (1979). National cooperative Crohn’s disease study: study design and conduct of the study. Gastroenterology.

[20] Schnitzler F, Fidder H, Ferrante M (2009). Mucosal healing predicts long-term outcome of maintenance therapy with infliximab in Crohn’s disease. Inflamm Bowel Dis.

[21] Ferrante M, Colombel JF, Sandborn WJ (2013). Validation of endoscopic activity scores in patients with Crohn’s disease based on a post-hoc analysis of data from SONIC. Gastroenterology.

[22] Modigliani R, Mary JY, Simon JF (1990). Clinical, biological, and endoscopic picture of attacks of Crohn’s disease. Evolution on prednisolone. Groupe d’Etude therapeutique des affections inflammatoires digestives. Gastroenterology.

[23] Landi B, Anh TN, Cortot A (1992). Endoscopic monitoring of Crohn’s disease treatment: a prospective, randomized clinical trial. The groupe d’Etudes therapeutiques des affections inflammatoires digestives. Gastroenterology.

[24] Cellier C, Sahmoud T, Froguel E (1994). Correlations between clinical activity, endoscopic severity, and biological parameters in colonic or ileocolonic Crohn’s disease. A prospective multicentre study of 121 cases. The groupe d’Etudes therapeutiques des affections inflammatoires digestives. Gut.

[25] Buisson A, Chevaux JB, Hudziak H (2013). Colonoscopic perforations in inflammatory bowel disease: a retrospective study in a French referral centre. Dig Liver Dis.

[26] Pariente B, Cosnes J, Danese S (2011). Development of the Crohn’s disease digestive damage score, the Lémann score. Inflamm Bowel Dis.

[27] Baumgart DC, Sandborn WJ (2012). Crohn’s disease. Lancet.

[28] Madsen SM, Thomsen HS, Schlichting P (1999). Evaluation of treatment response in active Crohn’s disease by low-field magnetic resonance imaging. Abdom Imaging.

[29] Ordas I, Rimola J, Rodriguez S (2014). Accuracy of magnetic resonance enterography in assessing response to therapy and mucosal healing in patients with Crohn’s disease. Gastroenterology.

[30] Van Assche G, Herrmann KA, Louis E (2013). Effects of infliximab therapy on transmural lesions as assessed by magnetic resonance enteroclysis in patients with ileal Crohn’s disease. J Crohns Colitis.

[31] Rutgeerts P, Van Assche G, Vermeire S (2006). Review article: infliximab therapy for inflammatory bowel disease–seven years on. Aliment Pharmacol Ther.

[32] Tielbeek JA, Lowenberg M, Bipat S (2013). Serial magnetic resonance imaging for monitoring medical therapy effects in Crohn’s disease. Inflamm Bowel Dis.

